# Partial RNA design

**DOI:** 10.1093/bioinformatics/btae222

**Published:** 2024-06-28

**Authors:** Frederic Runge, Jörg Franke, Daniel Fertmann, Rolf Backofen, Frank Hutter

**Affiliations:** Department of Computer Science, University of Freiburg, Freiburg 79110, Germany; Department of Computer Science, University of Freiburg, Freiburg 79110, Germany; Department of Computer Science, University of Freiburg, Freiburg 79110, Germany; Department of Computer Science, University of Freiburg, Freiburg 79110, Germany; Department of Computer Science, University of Freiburg, Freiburg 79110, Germany

## Abstract

**Motivation:**

RNA design is a key technique to achieve new functionality in fields like synthetic biology or biotechnology. Computational tools could help to find such RNA sequences but they are often limited in their formulation of the search space.

**Results:**

In this work, we propose *partial RNA design*, a novel RNA design paradigm that addresses the limitations of current RNA design formulations. Partial RNA design describes the problem of designing RNAs from arbitrary RNA sequences and structure motifs with multiple design goals. By separating the design space from the objectives, our formulation enables the design of RNAs with variable lengths and desired properties, while still allowing precise control over sequence and structure constraints at individual positions. Based on this formulation, we introduce a new algorithm, libLEARNA, capable of efficiently solving different constraint RNA design tasks. A comprehensive analysis of various problems, including a realistic riboswitch design task, reveals the outstanding performance of libLEARNA and its robustness.

**Availability and Implementation:**

libLEARNA is open-source and publicly available at: https://github.com/automl/learna_tools.

## 1 Introduction

RNA design describes the problem of generating RNA sequences with certain properties to fulfill desired functions (Hammer *et al.* 2019a). The design endeavors often start from carefully selected RNA fragments with known functions to combine these so-called motifs and achieve new functionality (Wachsmuth *et al.* 2012, Li *et al.* 2018, Nozawa *et al.* 2019). The designed construct is then evaluated in a wet laboratory experiment proofing the desired functionality. To save costs, however, computational methods are employed to reduce the number of initial candidates for experimental validation (Hammer *et al.* 2019a).

The theoretical design space for computational models is typically huge. Thus, restricting this space to a reasonable subspace can drastically speed up the search for good candidates. It is common knowledge that primary- and secondary-structure features are useful restrictions, while the design goals might vary depending on the particular application (Hammer *et al.* 2019a). To optimally support RNA practitioners, an algorithm should thus be able to process any primary- and secondary structure restrictions, efficiently explore large design spaces, consider different design goals, and provide as many candidates as needed, optimally with different lengths and compositions. We observe that current RNA design approaches do not fully satisfy these requirements. Algorithms that follow the inverse RNA folding formulation require *a priori* knowledge about the exact pairing scheme of the final structure and do not consider primary structure restrictions (Hofacker *et al.* 1994, Andronescu *et al.* 2004, Busch and Backofen 2006). While others are generally capable of including primary structure restrictions (Lorenz *et al.* 2011, Yao *et al.* 2024) and developed relaxations to the strict pairing scheme (Kleinkauf *et al.* 2015, Minuesa *et al.* 2021) these algorithms still require at least specific regions for base pairs and tasks of a fixed length. Recent sampling methods (Hammer *et al.* 2019b, Yao *et al.* 2021, 2024) can sample large amounts of candidates with desired properties even for multiple target structures but they are still limited to fully defined base pairing schemes and fixed lengths tasks. Similarly, approaches that design RNA sequences based on abstract shapes (Shapiro 1988, Giegerich *et al.* 2004) are not limited to a specific output length but require a fully defined secondary structure as a template to derive the shape (Avihoo *et al.* 2011, Retwitzer *et al.* 2016). This limitation of the search space appears suboptimal and impractical. For example, consider the design of constructs that contain a GNRA tetra-loop motif to establish tertiary interactions for subsequent crystallization studies (Reyes *et al.* 2009). Then one might want to design 50 candidate constructs with variable lengths, that contain a stem of unspecified length with a GNRA tetra-loop. This kind of task cannot be tackled with any current method. We are not even aware of any formulation that supports the definition of a task with a single paired position without defining its pairing partner, region, or an entire final shape because design approaches do not consider unbalanced pairing schemes.

In this work, we propose *partial RNA design*, a novel RNA design paradigm that tackles these issues. Our formulation separates the definition of the design space from the design objectives, allowing arbitrary sequence and structure restrictions of the design space. Using this problem definition, we enhance a previous RNA design approach (Runge *et al.* 2019) to develop *libLEARNA*, a new algorithm that can explore large RNA design spaces guided by sequence and secondary structure motifs to yield large amounts of variable length candidate constructs for different design objectives. Our main contributions are as follows:

- We propose *partial RNA design*, a novel RNA design paradigm formulated as a constraint satisfaction problem (CSP). Partial RNA design is more general than any previous RNA design formulation and allows the design of RNA sequences with different lengths while providing precise control over local and global restrictions of the sequence and structure space (Section 2).
- We improve an existing RNA design framework, LEARNA (Runge *et al.* 2019), with a masked training objective to develop *libLEARNA*, a new algorithm that is capable of efficiently navigating large RNA design spaces, ensuring the inclusion of desired motifs while exploring the space within the set parameters (Section 3).
- We evaluate *libLEARNA* on various RNA design tasks, demonstrating its strong performance and robustness across multiple search spaces and design objectives (Section 4).
- We open-source our RNA design framework at https://github.com/automl/learna_tools.

## 2 Partial RNA design

Formulations of secondary structure based RNA design can be divided into two major parts: the objectives that are used to rank the designed candidates and a specific search space. A well-known paradigm is inverse RNA folding where the goal is to design an RNA sequence $\phi \in \Phi^{*}:={\left\{A,C,G,U\right\}}^{*}$ that folds into a desired secondary structure $\omega \in \Omega^{*}:=\{.,),{\left(,\right\}}^{*}$, where ${\;}^{*}$ denotes the *Kleene closure* (Fletcher *et al.* 1991). The design objective, O, might vary (Hammer *et al.* 2019a): Hofacker *et al.* (1994) searched for a sequence $x\in \Phi^{*}$ whose minimal free energy (MFE) structure, $y\in \Omega^{*}$, is equal to the target structure; Zhou *et al.* (2023) searched for candidates whose secondary structure ensembles contain the target; Esmaili-Taheri and Ganjtabesh (2015) and Rubio-Largo *et al.* (2019) used a multi-objective approach. The search space, however, is consistently defined with a fixed length and specific (primary- and) secondary structure features at certain positions. A common formalization of this problem is a CSP (Garcia-Martin *et al.* 2013, 2015, Hammer *et al.* 2019b, Minuesa *et al.* 2021, Yao *et al.* 2024). CSPs are general formulations of combinatorial problems defined as a triple (X,D,C), where *X* is a set of variables, *D* is a set of their corresponding domains of values, and *C* is a set of constraints. Each variable can take on the values of its corresponding non-empty domain. A constraint c∈C is a tuple (t,R) with t⊆X being a subset of *k* variables from *X* and R being a *k*-ary relation over these variables. However, previous CSP formulations for modeling RNA design typically use the variables in *X* to assign nucleotides to individual positions of an RNA with a fixed length, given specific base pair constraints. The exact length or composition of the best candidate for solving a given design problem, however, is often unclear. For example, it is well known that certain structure motifs could increase the binding affinity of aptamers to a target, while the best length of the aptamer and the exact position of these motifs is typically not known (Vorobyeva *et al.* 2018).

### 2.1 The partial RNA design problem

In contrast to existing CSP formulations for RNA design, partial RNA design is defined as a search over RNA motifs rather than individual nucleotide positions of an RNA. We define two variables *x* and *y* to represent the designs of the primary- and secondary-structure parts of a motif, respectively. These variables can be assigned to any sequences of nucleotide and any sequences of secondary structure symbols and we consider a simultaneous search over both. The corresponding domains of values for *x* and *y* are defined as follows:

(1) $$\begin{array}{c} D_{x}:=\{\phi \;|\;\phi \in \Phi^{*}:={\left\{A,C,G,U\right\}}^{*}\}\\ \text{and}\\ D_{y}:=\{\omega \;|\;\omega \in \Omega^{*}:=\{.,),{\left(,\right\}}^{*}\}\;. \end{array}$$

Further, we introduce two wildcards that allow us to define search spaces of arbitrary length and composition. Formally, we extend the RNA nucleotide and secondary structure alphabets, Φ:={A,C,G,U} and Ω:={.,),(,}, with a *wildcard symbol* “?” indicating a position that is not constrained, and a *variable length wildcard symbol* “$\overset{*}{?}$” to indicate an unconstrained region:

(2) $$\begin{array}{c} \hat{\Phi }:=\Phi \cup \{?\}\cup \{\overset{*}{?}\}\\ \hat{\Omega }:=\Omega \cup \{?\}\cup \{\overset{*}{?}\}, \end{array}$$

A *motif* **m** then is a pair $\left(\hat{\phi },\hat{\omega }\right)$, consisting of an RNA sequence part $\hat{\phi }\in {\hat{\Phi }}^{*}$, and an RNA structure part $\hat{\omega }\in {\hat{\Omega }}^{*}$. For a simple target structure “((….))”, the search space for our inverse RNA folding task can then be described with a single motif m=(“????????”, “((….))”). However, we can also define more complex search spaces with multiple diverse motifs. For example, recall our example of designing RNAs that contain a GNRA tetra-loop. We can define a search space using three motifs: $m_{1}=(“\overset{*}{?}”,\;“\overset{*}{?}\;”)$ with two variable length wildcards to represent a region of arbitrary length and composition, a specific motif for the GNRA-loop $m_{2}=(“?\mathrm{GNRA}?”,\;“(\ldots .)”)$, and a second variable length motif to allow for extensions after the loop motif, $m_{3}=(“\overset{*}{?}”,“\overset{*}{?}\;”)$. Given this search space, we can define the set of nucleotide and structure sequences that are contained in it: For the first and last motif, we are free to assign any sequence of nucleotides and structure symbols, while we have a length limitation of six for both parts of the second motif. We can formalize this using two variables, $x_{i},y_{i},i\in \{1,2,3\}$, for each motif. However, the assignment of random sequences of nucleotides and secondary structure symbols to our variables is not task-specific and error-prone. We address this with the definition of objectives. An objective O(·) defines a relationship between a subset, t⊆X, of k=|t| many variables from *X*. The solution space for a given objective *O* is a set of tuples of m≤k many variables *x* and n=k−m many variables *y* that satisfy the objective. We can write this as a relation $R_{O}:\prod_{i=1}^{m}D_{x_{i}}\times \prod_{i=1}^{n}D_{y_{i}}$, with

(3) $$\begin{array}{c} R_{O}:=\{\left(x_{1},\ldots ,x_{m},y_{1},\ldots ,y_{n}\right)\\ |\;O(x_{1},\ldots ,x_{m},y_{1},\ldots ,y_{n})\;\text{is satisfied}\}. \end{array}$$

For example, to treat specific nucleotide and structure symbols provided with a motif $m=(\hat{\phi },\hat{\omega })$ as hard constraints, we can define the relation $R_{m}:D_{x}\times D_{y}$ with

(4) $$\begin{array}{c} R_{m}:=\{(x,y)\;|\;\forall i\in \{1,\ldots ,|\hat{\phi }|\},j\in \{1,\ldots ,|\hat{\omega }|\}:\\ ({\hat{\phi }}_{i}\in \Phi \Leftrightarrow x_{i}={\hat{\phi }}_{i})\wedge ({\hat{\omega }}_{j}\in \Omega \Leftrightarrow y_{j}={\hat{\omega }}_{j})\}. \end{array}$$

Setting *t *=* X*, we can further define our folding objective with a global folding relation $R_{F}:\prod_{i=1}^{k}D_{x_{i}}\times \prod_{i=1}^{k}D_{y_{i}}$ over all concatenated sequence parts, $x_{1}\circ \ldots \circ x_{k}$, that have to fold into the concatenation of the structure parts: $F(x_{1}\circ \ldots \circ x_{k})=y_{1}\circ \ldots \circ y_{k}$, with

(5) $$\begin{array}{c} {\mathbb{R}}_{F}:=\{\left(x_{1},\ldots ,x_{k},y_{1},\ldots ,y_{k}\right)\\ \;|\;F(x_{1}\circ \ldots \circ x_{k})=y_{1}\circ \ldots \circ y_{k}\}. \end{array}$$

Generally, we are free to define any objectives e.g. to only consider the sequence parts to define a GC-content objective. The *partial RNA design problem* can then be defined as follows.

Definition 1. **Partial RNA design problem.** Given a sequence of RNA motifs, $S=(m_{1},\ldots ,m_{k})$, of length k∈ℕ and a set of n∈ℕ objectives $O=\{O_{1},\ldots ,O_{n}\}$, a solution to the partial RNA design problem associated with the sequence S corresponds to a solution of the CSP (X,D,C), defined as follows:

$$X=\overset{k}{\underset{i=1}{\cup }}\left\{x_{i},y_{i}\right\},D=\overset{k}{\underset{i=1}{\cup }}\left\{D_{x_{i}},D_{y_{i}}\right\},C=\overset{n}{\underset{i=1}{\cup }}\left\{\left(t_{i}\subseteq X,R_{O_{i}}\right)\right\}.$$

The partial RNA design problem describes a completely novel RNA design paradigm: In contrast to previous work that tries to find optimal solutions in a very restricted setting, our approach allows to design RNAs that are contained in a broader design space, essentially designing RNA focus libraries while considering arbitrary primary- and secondary-structure constraints. We also note that we do not limit the definition of the motifs. For example, structure parts can contain any composition of brackets, balanced or unbalanced. This allows us to create structural diversity and further enables us to extend the task at any given point since we are no longer bound to explicit pairing positions. However, one can define an objective to mark specific positions that have to be paired. We observe that reinforcement learning provides a framework that resonates very well with partial RNA design because it separates the search space (via observed states) from the objective (the reward function) by definition. In the following section, we detail the development of, libLEARNA, an automated reinforcement learning (Parker-Holder *et al.* 2022) algorithm that is capable of designing RNAs from provided sequence and structure motifs under different objectives.

## 3 Materials and methods

In this section, we develop libLEARNA, a new algorithm capable of solving the partial RNA design problem. Partial RNA design can be interpreted as a masked prediction task, where the goal is to assign values to the unknown regions of a partially restricted RNA design space, similar to the masked training objective of e.g. the language model BERT (Devlin *et al.* 2018). For libLEARNA, we seek to learn the navigation of a partially restricted design space across thousands of tasks with fixed lengths. During evaluation, a task of variable length, i.e. a task that contains a variable length wildcard symbol $\overset{*}{?}$, is interpreted as a set of fixed length tasks. To solve it, we sample a fixed length task from the provided design space and try to solve it with a single shot. Since partial RNA design separates the objectives from the formulation of the design space, this strategy imposes three challenges: (i) Our algorithm has to be capable of navigating partially restricted RNA design tasks, (ii) it has to be able to adapt to changing objectives, and (iii) it requires strong performance with the first prediction. Before we dive into the details of how to achieve these goals, we briefly recap the foundation of libLEARNA, LEARNA (Runge *et al.* 2019).

LEARNA is an automated deep reinforcement learning (AutoRL) (Parker-Holder *et al.* 2022) algorithm for the generative design of RNA sequences, following an inverse RNA folding approach (Hofacker *et al.* 1994). The algorithm has two stages: offline training and optimization and online application. At offline training time, in an inner-loop, an agent learns an RNA design policy via periodical interactions with an environment. The agent receives a state from the environment and chooses an action based on the current state. The action is then communicated to the environment to compute a reward signal that informs the agent about the value of the chosen action. In LEARNA, states are defined as local representations of the input structure and actions correspond to placing a nucleotide or placing Watson–Crick pairs in case the structure indicates that the given position is paired. The reward is computed once all sites have been assigned nucleotides; after applying a folding algorithm to the designed sequence, the reward function is based on the Hamming distance between the folded candidate and the desired structure. The policy of the agent is defined through a neural network.

After training, still as part of the offline phase, the agent is evaluated on a validation set, and the loss is communicated to an efficient Bayesian optimization method, BOHB (Falkner *et al.* 2018), that jointly optimizes the configuration of the RL system in the outer loop, seeking to minimize the observed loss. During online application to a new test problem, the configuration that worked best on the validation set is then executed. Runge *et al.* (2019) propose three versions within this framework: (i) A version that is not trained at all but updates its policy during evaluation to adapt to a task at hand (LEARNA), (ii) a version that leverages the pretraining of the policy but fixes the weights during evaluation for fast sampling of candidates (Meta-LEARNA), and (iii) a pre-trained version that allows weight updates at evaluation time (Meta-LEARNA-Adapt). We use the Meta-LEARNA-Adapt approach to develop libLEARNA, because it leverages the advantages of a pre-trained policy while being able to adapt to a given task at hand.

### 3.1 A decision process for partial RNA design

A reinforcement learning problem can be defined as a Markov decision process D:=(S,A,R,P), with a set of *states* S, a set of *actions* A, a *reward function* R, and a *transition function* P. For our formulation, we consider a sequence of fixed length motifs $S=(m_{1},\ldots ,m_{n}),n\in \mathbb{N}$ with each motif $m_{i}=({\bar{\phi }}_{i},{\bar{\omega }}_{i}),i\in \{1,\ldots ,n\}$, consisting of a sequence part ${\bar{\phi }}_{i}\in {\bar{\Phi }}^{l_{i}}={\left\{A,C,G,U,?\right\}}^{l_{i}}$ and a structure part ${\bar{\omega }}_{i}\in {\bar{\Omega }}^{l_{i}}=\{.,),{\left(,?\right\}}^{l_{i}}$ of the same length *l_i_*. Note that, in contrast to $\hat{\phi },\;\bar{\phi }$ contains no variable length wildcard $\overset{*}{?}$. For simplicity, we define the task τ as the concatenation ° of the sequence and structure parts of the motifs: $\tau =(\bar{\phi },\bar{\omega })=({\bar{\phi }}_{1}{}^{\circ}\ldots {}^{\circ}{\bar{\phi }}_{n},{\bar{\omega }}_{1}{}^{\circ}\ldots {}^{\circ}{\bar{\omega }}_{n})$.

At each time step t=0,1,2,…,T, where *T* denotes the terminal time step of the episodic interaction between the agent and the environment, the environment provides a state $s^{t}\in S$. The agent chooses an action $a^{t}\in A$ based on the provided state and the environment transitions into a new state $s^{t+1}$ based on the chosen action, following the transition dynamics P. At the final time step *T*, the environment computes a scalar reward defined by the reward function R that is communicated to the agent to guide the learning of a policy. For libLEARNA, the states correspond to local representations of the provided task and actions correspond to placing nucleotides. To provide states, the environment transitions over local representations at unconstrained sites of the sequence part of the task. The length *T* of the interaction between the environment and the agent thus depends on the provided motifs in S. The reward during training is based on the number of satisfied constraints of the structure part of the task. In the following, we detail the individual components of the decision process D.

#### 3.1.1 State space

While Runge *et al.* (2019) only consider structure features for the state, we consider primary and secondary structure features for libLEARNA. To enable libLEARNA to process sequence and structure information, we numerically encode pairs of a sequence symbol and its corresponding structure symbol for each position of a given design task by indexing all pairs in the set $\left(\bar{\Phi }\times \bar{\Omega }\right)\cup \{(\#,\#)\}$. We provide local information about the task to the agent by setting the state *s^t^* to the (2κ+1)-gram centered around the position of the numerical vector representation of the task τ. Since we only consider unconstrained regions, *t* corresponds to the *t*th unconstrained position (denoted with ?) of the sequence part $\bar{\phi }$. κ is a hyperparameter dubbed the *state radius*. To be able to construct the centered n-gram at all sites, κ padding characters (“#”) are introduced at the start and the end of $\bar{\phi }$ and $\bar{\omega }$. Formally, the state space can be written as

(6) $$\begin{array}{c} S:=\{x^{\kappa }yz^{\kappa }\;|\;\\ x,z\in \left(\left(\bar{\Phi }\times \bar{\Omega }\right)\cup \{(\#,\#)\}\right),y\in \{?\}\times \bar{\Omega }\}. \end{array}$$

Besides the hyperparameter *κ*, we define a parameter σ that decides whether previous actions contribute to the current state or not: If σ is set to true, at each time step *t *>* *0, the last placed nucleotide replaces the respective wildcard in the nucleotide constraints to inform the agent about its actions. The numerical representation of the task is then updated at each step accordingly.

#### 3.1.2 Actions space

In each episode, the agent designs an RNA sequence $\phi \in \Phi^{\left|\bar{\phi }\right|}$, that satisfies all constraints of the sequence part $\bar{\phi }$. To design a candidate solution the agent places nucleotides by choosing an action *a^t^* at each time step *t* to fill the sites that are unrestricted in $\bar{\phi }$. Since an RNA sequence consists of four nucleotides (A, C, G, and U), the action space is formulated as A:={0,1,2,3}≡{A,G,C,U}. However, we define the *action semantics* hyperparameter that, if active, leverages knowledge about paired sites by directly placing Watson–Crick base pairs (AU, UA, GC or CG) as proposed by Runge *et al.* (2019). Note that we do not enforce balanced brackets. For an opening bracket, the site of the corresponding closing bracket thus might not be known. In this case, a single nucleotide is placed for an opening bracket regardless of the choice for the *action semantics* parameter.

#### 3.1.3 Reward function

The reward function implements the set of objectives O of our formulation of partial RNA design (see Definition 1). To be able to navigate large RNA spaces, we need to learn a reasonable relationship between the sequence space and the structure space. The folding relation from Equation (5) appears as an objective that relates both in a meaningful way. Therefore, we focus on learning to generate sequences that fold into a structure that satisfies all structure constraints during training and leverage this knowledge at evaluation time when designing RNAs for different objectives. We use RNAFold (Lorenz *et al.* 2011) with minimum free energy (MFE) structure predictions for folding of the candidate sequences during training.

At the terminal time step *T*, the agent has assigned nucleotides to all sites, and the environment computes the reward $R^{T}$. The reward is computed as the number of structure constraints that are satisfied, thus, unconstrained sites are ignored. Formally, we define the structure-loss $L_{\bar{\omega }}$ as

(7) $$L_{\bar{\omega }}:={\text{d}}_{H}{\;}_{\bar{\omega }}(\bar{\omega },F_{\text{MFE}}(\phi ))$$

with

(8) $${\text{d}}_{H}{\;}_{\bar{\omega }}(\bar{\omega },F_{\text{MFE}}(\phi ))=\sum_{i=1}^{\left|\;\bar{\omega }\;\right|}\;1_{\Omega }\left({\bar{\omega }}_{i}\right)\cdot d_{H}\left({\bar{\omega }}_{i},F_{\text{MFE}}{\left(\phi \right)}_{i}\right),$$

where $\bar{\omega }$ is the fixed length structure part of the task τ, $1_{\Omega }$ is the indicator function that returns 0 if the *i*th position of $\bar{\omega }$ is unrestricted, ${\bar{\omega }}_{i}=?$, and 1 if ${\bar{\omega }}_{i}\in \Omega \equiv \{.,),(\}$, and ${\text{d}}_{H}(\cdot ,\cdot )$ is the Hamming distance. We normalize this loss by the length of $\bar{\omega }$ to formulate the reward $R^{T}$:

(9) $$R^{T}:={\left(1-\frac{L_{\bar{\omega }}}{\left|\bar{\omega }\right|}\right)}^{\alpha },$$

We note again that the reward function depends on the objective and we change the reward function at evaluation time accordingly (see Section 4).

#### 3.1.4 Transition dynamics

At each time step *t*, the state is set to a fixed (2κ+1)-gram centered around the position in the numerical representation of the task that corresponds to the *t*th unconstrained position of the sequence part $\bar{\phi }$. Subsequent states are defined by transitioning to the next unconstrained position. Depending on the choices of the action semantics and the state composition, the transition dynamics may vary and are implemented accordingly.

### 3.2 Training regimen

We generate three training datasets from all sequences of the Rfam (Griffiths-Jones *et al.* 2003) database version 14.1 with different length distributions (≤200 nucleotides (nt), ≥200 nt, random length) of 100 000 samples each, and a non-overlapping validation set of 100 samples using *RNAFold* (Lorenz *et al.* 2011) to receive the structures. We detail our data pipelines in Supplementary Section SA and describe all datasets in Supplementary Table S1. The choice of the training dataset is a hyperparameter. We use a masked training objective similar to the masked language model training in BERT (Devlin *et al.* 2018). The task of *libLEARNA* during training is to fill the masked parts of the sequence such that, after folding the designed sequence, all positions of the folding satisfy the positional constraints of the masked structure. To derive partially restricted tasks, we first mask up to five parts of the structures, each covering up to 20% of the total length, while the positions, the lengths, and the number of parts are sampled uniformly at random. In the second step, we mask corresponding parts of the sequences that remain unmasked in the structures to derive tasks of alternating sequences and structure constraints. Finally, we randomly mask the sequences of ∼20% of the samples to derive tasks that correspond to RNA design from arbitrary sequence and structure motifs. Examples of the resulting training tasks are shown in Table 1.

**Table 1.** Examples of different tasks in the training data.

Description	Sequence	Structure	Proportion (%)
Inverse RNA folding	?????????	(((…)))	11.5
Alternating constraints	??GACU??C	((????))?	66.7
Random masking	G???CUC??	??(..???)	21.8

### 3.3 Automated reinforcement learning

Hyperparameters in reinforcement learning (RL) are known to be sensitive (Henderson *et al.* 2018). We, therefore, use an AutoRL approach similar to Runge *et al.* (2019) to automatically find the best RL system for our task in a single algorithm run. Please see Supplementary Fig. S1 for an outline of the general optimization procedure.

We mainly adopt the configuration space proposed by Runge *et al.* (2019) but introduce four new dimensions: The *action semantics* parameter to decide whether to predict single nucleotides only or Watson–Crick pairs at paired position, the *individual state composition* parameter to decide if the agent’s actions contribute to the state, as well as two choices that decide about the training data and its schedule. We further allow searching over an additional LSTM layer. The result is an 18-dimensional search space to jointly optimize over the network architecture, all parts of the decision process, as well as training hyperparameters, task distributions, and their schedule, using BOHB (Falkner *et al.* 2018). Details about the hyperparameter optimization can be found in Supplementary Section SB.3. The configuration space, hyperparameters, and the priors we use over them, as well as the final configuration of libLEARNA are shown in Supplementary Table S2. We use the exact same setup during meta-optimization as Runge *et al.* (2019), with the same training budgets and validation protocols. The final model of libLEARNA is thus only trained once on the full training budget of 1 h on 20 cores of a Broadwell E5-2630v4 2.2 GHz CPU with 5GB RAM per core with asynchronous policy updates. Please find more details on the model selection and training process in Supplementary Sections SB.1 and SB.2 as well as Supplementary Fig. S2. However, while Runge *et al.* (2019) optimize for an RL algorithm without any policy updates at test time, we directly optimize for an algorithm with policy updates at evaluation time to increase the adaptation capabilities of our approach.

## 4 Experiments

In this section, we show that our proposed strategy is reasonable by demonstrating libLEARNA’s strong performance with the first prediction, its ability to navigate partially restricted design spaces, and its capabilities to adapt to different objectives on tasks with a fixed length (Section 4.1). We then apply libLEARNA to different partial RNA design tasks that require navigation of large design spaces under different objectives (Section 4.2). We use the same model of libLEARNA, obtained from a single meta-optimization run, for all experiments without retraining or changing any hyperparameters.

### 4.1 Validation of strategy

#### 4.1.1 Improved one-shot performance

**Figure 1.**
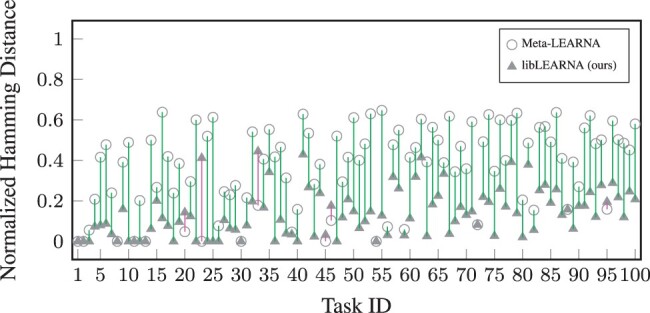
Comparison of the first predictions of Meta-LEARNA and libLEARNA on all tasks of the Eterna100 benchmark version 2. The plot shows the average normalized Hamming distance of the first prediction for all tasks of the benchmark. Green bars indicate tasks where libLEARNA achieves lower Hamming distance, and purple bars indicate tasks where the Hamming distance is above that of Meta-LEARNA’s prediction.

To analyze the quality of the first predictions of libLEARNA, we compare it to its predecessor with the strongest performance, Meta-LEARNA (Runge *et al.* 2019), on the commonly used Eterna100 benchmark version 2 (Koodli *et al.* 2021). The results are shown in Fig. 1. We observe that libLEARNA clearly outperforms Meta-LEARNA, showing a lower Hamming distance for ∼95% of the tasks. We also compare libLEARNA’s performance to SAMFEO (Zhou *et al.* 2023) and find that libLEARNA outperforms SAMFEO in terms of first predictions, while its first predictions perform only slightly worse than SAMFEO after one full iteration of optimization (see Supplementary Figs S3 and S4). We take this as an indicator that libLEARNA is generally capable of solving tasks with the first prediction.

#### 4.1.2 Navigating partially restricted design spaces

**Figure 2.**
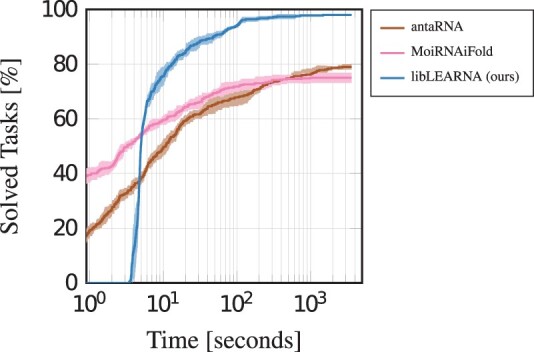
Comparison on randomly masked tasks with balanced brackets.

To assess libLEARNA’s performance on fixed length partially restricted tasks, we create a new dataset from the Rfam database version 14.1 and compare libLEARNA against antaRNA (Kleinkauf *et al.* 2015) and MoiRNAiFold (Minuesa *et al.* 2021), two algorithms that are capable of predicting RNAs from masked structures of fixed length. Due to a lack of publicly available RNA benchmarks for masked predictions, we randomly select a total of 100 sequences and randomly mask parts of the sequences and the structures after folding with ViennaRNA’s RNAFold (Lorenz *et al.* 2011). We ensure that sequences and structures are disjoint from the training and validation data and balance all brackets to account for the requirements of antaRNA and MoiRNAiFold. A task counts as solved if all constraints in the sequence and the structure are satisfied, ie multiple candidates are valid for a given input task. We run each algorithm for one hour on each task of the benchmark in five independent runs and report the average number of solved tasks with the standard deviation around the mean. The results are shown in Fig. 2. We observe that libLEARNA clearly outperforms antaRNA and MoiRNAiFold, indicating that our training strategy enabled libLEARNA to efficiently use the restrictions to find good regions in the design space.

#### 4.1.3 Robustness to changes of the objective

The training objective of libLEARNA has three components: a folding algorithm for folding the candidate solution, a distance measure to quantify the difference between the folded candidate and the desired structure constraints (see Equation (7)), and a reward function that uses the structure loss to define a reward signal (see Equation (9)). To analyze the behavior of libLEARNA to changes in the objective, we change one component at a time.

##### 4.1.3.1 libLEARNA adapts to changes of the loss function

To analyze the adaptation to changes of the loss function, we either change the distance measure, the folding algorithm, or both. libLEARNA is based on the Meta-LEARNA-Adapt approach (Runge *et al.* 2019) which uses a restart option that resets the weights of the policy network to the initial values every 1800 s. However, some changes might require a longer time for adaptation. We, therefore, evaluate each variant with and without the restart option. We again use the Eterna100 benchmark version 2 for evaluation. For the distance metric, we replace the Hamming distance with a Weisfeiler–Lehman (WL) graph kernel as recently proposed for the evaluation of secondary structure prediction (Runge *et al.* 2023). This change affects Equation (8) by replacing the Hamming distance ${\text{d}}_{H}$ with a WL distance measure $d_{\text{WL}}=(1-S_{\text{WL}}(G_{1},G_{2}))$, where *G*_1_ and *G*_2_ are the graphs of the secondary structures and $S_{\text{WL}}(G_{1},G_{2})=\psi (G_{1})\cdot \psi (G_{2})$, with $\psi (G1)\cdot \psi (G_{2})$ representing the dot product of the feature vectors of the graphs obtained through iterative aggregation of their labels via hash functions. For the folding engine, we use the RNAFold variant for the maximum expected accuracy (MEA) structure instead of the MFE structure, i.e. we replace the folding algorithm $F_{\text{MFE}}$ with $F_{\text{MEA}}$ in Equation (7). The accumulated number of solved tasks across all runs for each variant is shown in Table 2. Surprisingly, changing the distance measure results in better performance compared to the original libLEARNA baseline with and without restarts of the algorithm. However, for the folding engine, we observe that the change to the MEA folding objective decreases the performance in the setting with restarts, while it seems to be beneficial when there is no restart performed, independent of the distance measure. We conclude that changing the folding engine is a critical change to the objective that requires more time for adaptation. This is also in line with recent work that showed that learning-based approaches for RNA design often struggle with changes to the folding algorithm (Koodli *et al.* 2021). However, overall the performance of libLEARNA appears robust to changes in the loss function.

**Table 2.** Analysis of changes applied to the loss function.

Variation	Solved	
	Restart	No restart
libLEARNA	76	72
libLEARNA-WL	77	73
libLEARNA-MEA	75	78
libLEARNA-MEA-WL	71	78

##### 4.1.3.2 libLEARNA adapts to changes of the reward function

To analyze the influence of a change on the reward function, we evaluate libLEARNA when adding a GC-content objective. We implement an additional GC-loss term in the reward function. The GC-loss, *L*_GC_, is defined as the absolute deviation of the GC-content of the designed sequence $\phi ,\;{\text{GC}}_{\phi }$, from the desired GC-content, ${\text{GC}}_{\text{desired}}$, allowing a given tolerance ϵ:

(10) $$L_{\text{GC}}:=\{\begin{array}{cc} 0 & \text{if}\;|{\text{GC}}_{\text{desired}}-{\text{GC}}_{\phi }|\leq \epsilon \\ \left|{\text{GC}}_{\text{desired}}-{\text{GC}}_{\phi }\right| & \text{else} \end{array}.$$

We use a tolerance ϵ=0.01 for all experiments with desired GC-contents. For the reward function $R^{T}$ we then use the weighted sum of the structure-loss and the GC-loss:

(11) $$R^{T}:=\{\begin{array}{cc} 0 & \text{if}\;\beta \cdot \frac{L_{\bar{\omega }}}{\left|\bar{\omega }\right|}+\gamma \cdot L_{\text{GC}}>1\\ {\left(1-\left(\beta \cdot \frac{L_{\bar{\omega }}}{\left|\bar{\omega }\right|}+\gamma \cdot L_{\text{GC}}\right)\right)}^{\alpha } & \text{else} \end{array}.$$

We set β=γ=1 without further tuning and note that libLEARNA was not trained to include desired GC contents. Runge *et al.* (2019) propose a local improvement step (LIS) to aid the agent in solving a task once it is close to a solution. We adopt this procedure and additionally implement a GC-improvement step (GIS) that becomes active whenever the LIS is active. We describe the GIS in more detail in Supplementary Section SC. We evaluate libLEARNA against antaRNA (Kleinkauf *et al.* 2015) and MCTS-RNA (Yang *et al.* 2017) on all pseudoknot-free tasks of the ArchiveII dataset (Sloma and Mathews 2016), a commonly used and well-prepared RNA benchmark set, using a timeout of one hour. We report the accumulated number of solved tasks across five independent runs. Table 3 shows the results. Remarkably, libLEARNA is on par with MCTS-RNA while clearly outperforming antaRNA. This confirms our previous observation that libLEARNA can adapt to changes in the objective.

**Table 3.** Results on tasks with desired GC-contents of the ArchiveII dataset.

Model	Solved (%)
antaRNA	52
MCTS-RNA	73
libLEARNA (ours)	73

### 4.2 Solving the partial RNA design problem

In this section, we tackle the problem of RNA design for partially restricted design spaces of variable length, essentially solving the partial RNA design problem with varying objectives. We design theophylline riboswitch constructs to assess libLEARNAs ability do design functional constructs using the training objective described in Equation (9) and the combined objective to design RNAs with desired GC-contents described in Equation (11), before we assess the performance of libLEARNA under completely new objectives.

#### 4.2.1 Automated design of theophylline riboswitches

In this section, we generate diverse, variable-length theophylline riboswitch constructs in a single run of libLEARNA, following a previously published protocol (Wachsmuth *et al.* 2012). Originally, Wachsmuth *et al.* (2012) constructed theophylline riboswitch candidates for transcriptional activation from (i) the TCT8-4 theophylline aptamer sequence and structure, (ii) a spacer sequence of 6–20 nucleotides (nt), (iii) a sequence of 10–21 nt complementary to the 3′-end of the aptamer, and (iv) a U-stretch of 8 nt at the 3′-end of the construct. To generate candidate sequences, Wachsmuth *et al.* (2012) create a large library of random sequences for the spacer region (6–20 nt) and a library of sequences complementary to the 3′-end of the aptamer (10–21 nt). From these sets, randomly sampled sequences were combined with the aptamer and the 8-U-stretch.

For the creation of the search space, we use the shared sequence and structure motifs of the six proposed riboswitch constructs by Wachsmuth *et al.* (2012) and combine them into a single design space formulation. The final definition of the design space and the proposed constructs of Wachsmuth *et al.* (2012) are shown in Supplementary Table S3.

We assess the performance of libLEARNA against the original library generation procedure proposed by Wachsmuth *et al.* (2012) in two experiments: (i) The design of a riboswitch library based on sequence and structure constraints only, and (ii) the design of sequences when additionally querying libLEARNA to design candidates with a specific GC-content, given a tolerance of 0.01. For each experiment, we generate 50 000 candidates with the approach of Wachsmuth *et al.* (2012) and libLEARNA with five random seeds and evaluate them using the same protocol as used by Wachsmuth *et al.* (2012) without ranking the constructs via z-scores.

**Figure 3.**
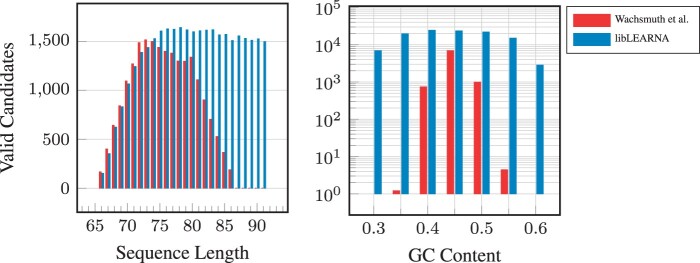
Average number of designed riboswitches across different lengths and GC-contents.

We observe that libLEARNA generates considerably more candidates that pass the design criteria compared to the original procedure proposed by Wachsmuth *et al.* (2012), yielding 71% satisfying candidates on average, compared to 42% for the original procedure (Table 4). Further, the candidates are nearly uniformly distributed across the lengths of the design space, especially for the longer sequences (Fig. 3 left), and the structure diversity generated by libLEARNA is around 23% higher (Table 4). When designing candidates with desired GC contents, libLEARNA provides up to 47% more candidates that satisfy the design criteria and the desired GC content (Fig. 3). Remarkably, libLEARNA can also design candidates on the margins of possible GC contents (0.3 and 0.6) which is between 0.29 and 0.63 for the given riboswitch design space.

**Table 4.** Overview of designed riboswitch candidates.

Method	Valid candidates (%)	Unique structures
Wachsmuth *et al.* (2012)	41.7	6316.6
*libLEARNA*	70.9	8269.6

#### 4.2.2 libLEARNA adapts to new objectives

**Figure 4.**
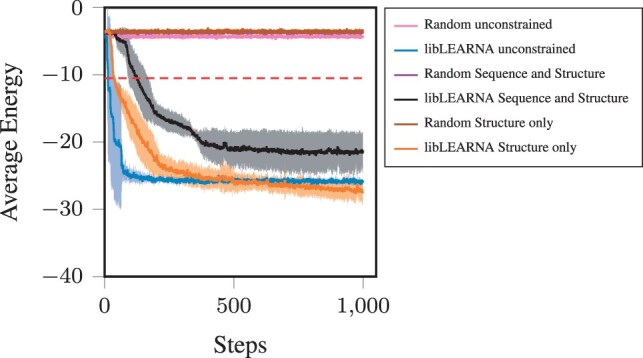
The average energy of RRI complex. A step in the figure corresponds to 500 episodes. The values are averages across five independent runs with standard deviation around the mean displayed as confidence bounds. The dashed red line indicates the energy level of the template sequence in complex with the target RNA.

Partial RNA design allows the definition of arbitrary objectives. We, therefore, assess libLEARNA’s performance on two objectives that are independent of a folding algorithm. In the first experiment, we seek to design RNAs that belong to the family of Hammerhead ribozymes (Type III) (Rfam family: RF00008). To guide the search, we use the consensus structure and the conserved nucleotides provided by the Rfam database while allowing for exploration at the beginning, end, and in the loop regions of the structure. We use the covariance model of the family (CLEN 54) to receive bitscores for each design, ϕ, using Infernal (Nawrocki and Eddy 2013) and design candidates with a total length between 50 and 60 nucleotides. We implement a new reward function that directly optimizes for bitscores: $R^{T}=\text{bitscore}(\phi )$. The reward for a candidate that does not match the covariance model at all is set to –200. We compare libLEARNA with a random agent that uniformly samples actions at each unconstrained position of the search space. The results are shown in Supplementary Fig. S5. libLEARNA quickly optimizes the bitscore to roughly 30 on average, while the average bitscores of the random agent remains below zero. For reference, we sample 1000 sequences directly from the covariance model using cmemit of the Infernal package. The highest achieved bitscore of these sequences is 71.1 compared to 72.2 when designing sequences with libLEARNA. However, the search space definition might affect the performance. We, therefore, analyze the influence of the search space definition in a second experiment where we use libLEARNA to design candidates for RNA-RNA interactions (RRI). More precisely, we design candidate sequences for a given target RNA that minimize the energy of the interaction obtained from IntaRNA (Mann *et al.* 2017). We use two RNAs of *Prochlorococcus marinus*: an mRNA target (GenBank Accession: BX548174; Region: 1069333.1069507) and an ncRNA (GenBank Accession: NC_008817; Region: 1002095.1002151) as template for the search. We define (i) A completely unrestricted search space that only contains a variable length wildcard symbol such that libLEARNA designs sequences guided solely by the reward function. (ii) A search space that only restricts the structure part; we fold the example sequence and introduce a point of extension (a variable length wildcard symbol $\overset{*}{?}$) in the middle of the resulting two hairpins. (iii) A search space with restrictions in the structure and the sequence part, by providing 10 nucleotides from the original sequence at random positions in addition to the search space defined in Equation (2). We implement a new reward function that optimizes for the inverse of the energy: $R^{T}={\left(\text{Energy}\cdot (-1)\right)}^{3}$. Again we compare libLEARNA to its random variant in five runs for 500 000 episodes. Figure 4 shows the results of the RRI design task while the energy is averaged across 500 steps and all runs. We observe that libLEARNA can design candidates with a lower energy of the complex compared to the random agent and the initial example sequence (indicated by a red line in Fig. 4). Interestingly, libLEARNA quickly adapts to the new reward function even without any sequence and structure information (unconstrained). However, the structure information seems to be beneficial in the long run since the final energy for the designed candidates using the design space with the structure information only is the lowest of all three search space formulations. The search space that contains sequence and structure restrictions seems to impose more challenges to the design process. While the resulting candidates still clearly show a lower energy with the target RNA compared to the original sequence, the optimization is slower and results in a higher final energy. Overall, we conclude that the formulation of the search space can have a substantial impact on the outcome of a given design task.

## 5 Conclusion

In this work, we propose *partial RNA design*, a new RNA design paradigm formulated as a CSP. The idea is to treat RNA design as a search in a partially restricted design space that can be explored by a machine under different objectives. We then propose libLEARNA, a robust AutoRL algorithm, capable of navigating partially restricted design spaces. We show that libLEARNA has strong performance across a wide range of different RNA design tasks, including restrictive design settings with fixed lengths and tasks that require the exploration of open design spaces and the generation of variable length candidates. Our work describes a novel approach to RNA design where the algorithm provides large amounts of solutions, given the restrictions of its search space rather than designing one or few solutions for a very narrow task definition. We believe that our new approach adds a new dimension to the RNA design problem and that libLEARNA is a useful tool for future RNA design endeavors.

## Supplementary Material

btae222_Supplementary_Data

## Data Availability

The data used in this work is available on GitHub at https://github.com/automl/learna_tools
