# Supplementary material for: Partial RNA design
Source: Bioinformatics. 2024 Jun 28;40(Suppl 1):i437–45. doi: 10.1093/bioinformatics/btae222 (PMC11256918; doi:10.1093/bioinformatics/btae222)
Supplement: btae222_Supplementary_Data [file btae222_supplementary_data.zip › btae222_Supplementary_Data/Runge.291.sup.1.pdf]

Supplementary Material for: Partial RNA Design

Frederic Runge[1], Jörg Franke[1], Daniel Fertmann[1], Rolf Backofen[1], Frank Hutter[1]

[1]Department of Computer Science, University of Freiburg

A Data

Initial Data Preparation We process our initial data as follows: We first download all sequences from 3016 families of the Rfam database [1] version 14.1. Each sequence was folded using RNAfold [2], resulting in an initial data pool of 6,370,459 RNA sequences with corresponding foldings. We dropped all structure duplicates to obtain 1,096,351 unique RNA secondary structures with corresponding unique sequences. From this data pool, we sample datasets without replacement uniformly at random: Three training sets of 100,000 samples each with different length distributions ($\leq$ 200 nucleotides, $\geq$ 200 nucleotides, random length), a disjoint validation set of 100 samples as well as a disjoint test set of 100 samples. These datasets were then masked using the proposed masking procedure described in the main paper.

Benchmark Preparation For benchmarking on the ArchiveII dataset [3], we remove all pseudo-knotted samples and ensure that there is no sequence nor structure overlap with the samples from the training and validation data. The Eterna100 benchmark version 2 [4] was used as is. However, we also checked all sequences and structures to ensure that this set is also disjoint from all training and validation sets.

Table S1: Overview over the Datasets.

**Data Set**	**Tasks**	**Mean Length**	**Median Length**	**Min/Max Length**
Training1 (“long”)	100000	470.9	300	200–8033
Training2 (“short”)	100000	103.9	99	23–200
Training3 (“random”)	100000	142.7	106	23–6361
Validation	100	135.8	94	46–1802
Rfam Testset	100	130.8	107	45–417
ArchiveII	282	135.7	118	30–708
Eterna100_v2	100	159.6	104	12–400

B Meta Optimization

libLEARNA follows an automated reinforcement learning approach where we iteratively optimize the configuration of the entire algorithm in a single run. The result of the meta-optimization is a fully trained agent with a specific environment and a set of tuned hyperparameters. At each iteration, we sample a configuration, train a reinforcement learning algorithm defined by this configuration on one out of three training data distributions (defined by the configuration) and evaluate it on a hold out validation set. For optimization, we use BOHB [5], a strong Bayesian Optimization method, that seeks to minimize the validation loss over time by sampling better configurations in each iteration.

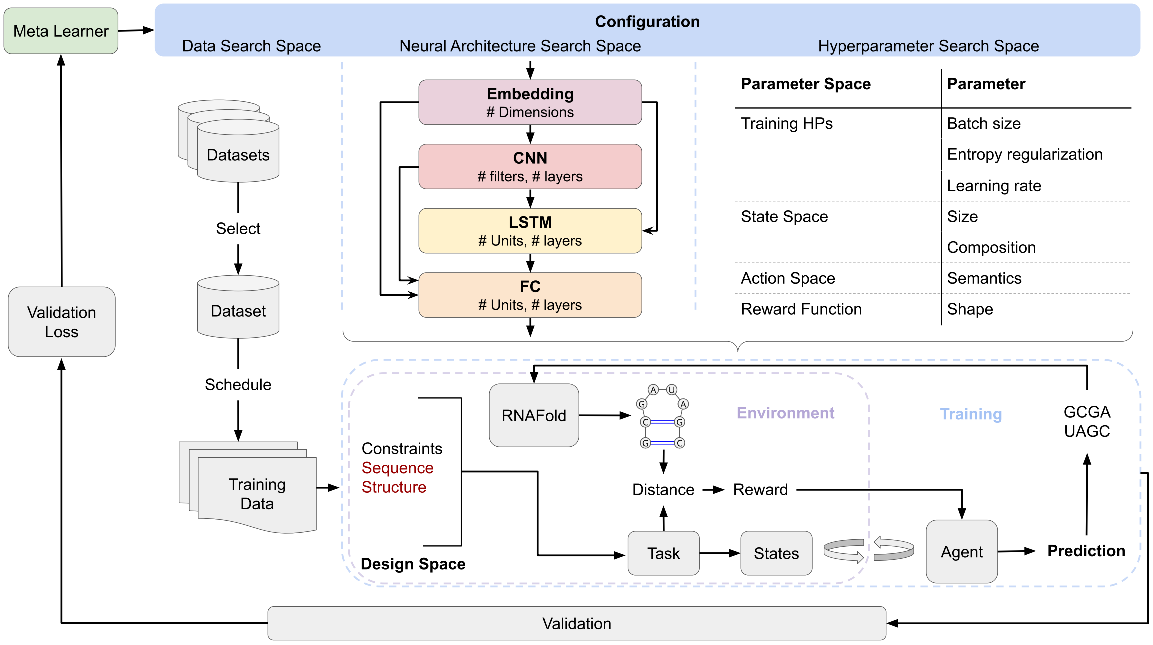

Figure S1: Meta-optimization loop. In each iteration, the meta-learner (BOHB) samples a configuration from a rich configuration space. The sampled configuration defines the learning algorithm's hyperparameters, a specific environment, a particular network architecture of the agent, a training curriculum, and a training data set. These components together formulate the learner; a deep reinforcement learning algorithm, which is trained on the selected training set and evaluated on a validation set. The resulting validation loss is communicated to the meta-learner to update its model, seeking to learn to sample better configurations with each iteration.

We run BOHB for 512 iterations with a maximum training budget of one hour using the training budget as a fidelity. The validation set contains a total of 100 samples and the evaluation budget was set to 60 seconds per sample. The general setup is exactly the same as used for LEARNA [6] to obtain a comparable model, except for four additional hyperparameters, slight adaptations to the hyperparameter ranges, and the masking of the training data. Please find an overview of the meta-optimization process in Figure S1.

B.1 Model Selection

We evaluate a total of 3415 configurations during our meta-optimization run for libLEARNA. From these, 1538 configurations were trained on a budget of 400 seconds, 1026 were evaluated after a training time of 1200 seconds, and 851 configurations were evaluated after the full training budget of 3600 seconds. In accordance to Runge et al. [6], we select the best configuration based on the number of solved tasks during validation, while we optimize the mean of the minimal hamming distances achieved per validation sample during meta-optimization. Figure S2 gives an overview of the validation performance of each configuration during meta-optimization.

B.2 Training Details

The final model of libLEARNA is trained only once during meta-optimization for one hour on 20 cores of Broadwell E5-2630v4 2.2 GHz CPUs with 5GB RAM per core using asynchronous policy updates. There is no GPU training involved and libLEARNA is only not retrained for any experiments. We further do not change any of the hyperparameters throughout our experiments.

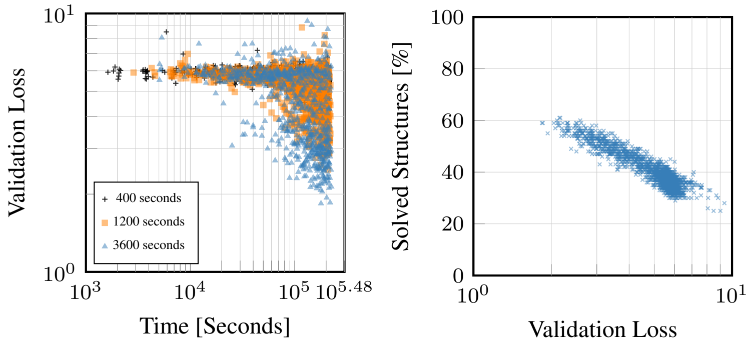

Figure S2: Overview of validation performance of configurations during meta-optimization. Left: Summary of the validation loss of all configurations. We observe that the validation loss of the configurations decreases constantly over the time. The plot implicates that stopping the meta-optimization earlier might have resulted in a worse model. However, we do not want to speculate that a longer optimization might have resulted in a better model. Right: Comparison of the validation loss with the model selection criteria of the number of solved validation tasks. The plot indicates that the validation loss is well correlated with our final model selection criteria.

B.3 Hyperparameters

The hyperparameters of libLEARNA are jointly optimized together with the architecture of the policy network, the choice of the training data and the task schedule. All parameters are only tuned once and we do not change any hyperparameters for all experiments. Please find a list of all hyperparameters, their ranges as well as the priors we used over them, and the finally selected configuration of libLEARNA in Table S2.

Table S2: Configuration space and final parameters of libLEARNA.

**Parameter Name**	**Type**	**Range**	**Prior**	**libLEARNA**
state radius κ	integer	$[0,\ 32]$	uniform	10
individual state composition σ	categorical	[“target”, “design”]	uniform	“target”
action semantics	categorical	[“pair”, “single”]	uniform	“pair”
reward exponent α	float	$[1,\ 12]$	uniform	10.76
filter size in 1st conv layer	integer	$\{0\} \cup \{3, 5, \dots, 17\}$	uniform	0
filter size in 2nd conv layer	integer	$\{0, 3, 5, 7, 9\}$	uniform	0
# filter in 1st conv layer	integer	$[1,\ 32]$	log-uniform	17
# filter in 2nd conv layer	integer	$[1,\ 32]$	log-uniform	24
# LSTM layers	integer	$[0,\ 3]$	uniform	0
# units in every LSTM layer	integer	$[1,\ 64]$	log-uniform	20
# fully connected layers	integer	$[1,\ 2]$	uniform	2
# units in fully connected layers	integer	$[8,\ 64]$	log-uniform	12
embedding dimensionality	integer	$[0,\ 21]$	uniform	17
batch size	integer	$[32,\ 256]$	log-uniform	247
entropy regularization	float	$[1 \cdot 10^{-7},\ 1 \cdot 10^{-2}]$	log-uniform	$4.46 \cdot 10^{-7}$
learning rate for PPO	float	$[1 \cdot 10^{-6},\ 1 \cdot 10^{-3}]$	log-uniform	$5.9 \cdot 10^{-4}$
training data	categorical	[“random”, “short”, “long”]	uniform	“short”
training curriculum	categorical	[“random”, “sorted”]	uniform	“sorted”

C Local Improvement Steps

Runge et al. [6] propose a local improvement step (LIS) that exhaustively evaluates neighboring sequences once the agent is close to a solution. We kept the LIS cut-off parameter ξ unchanged as proposed at Runge et al. [6], setting it to 5 which corresponds to at most $4^4 = 256$ neighboring sequences that are explored during LIS. For our experiments of RNA design with desired GC-contents we develop a similar GC-improvement procedure (GIS). The GIS becomes active whenever the LIS is active and is applied after the LIS. For the GIS we iteratively select a random nucleotide without replacement, while the respective choice depends on the current GC-content of the candidate sequence and the desired GC-content (either A or U if the GC content is too low or G and C if the GC-content is too high). We then replace the nucleotide with a randomly chosen nucleotide to either increase or decrease the GC-content. At each iteration, we fold the sequence and evaluate the structure-loss to keep it at least as low as at the beginning of the GIS. This procedure is repeated until no nucleotides are left for replacement. We provide pseudocode of the GIS in Algorithm 1.

Algorithm 1: GC Improvement Step (GIS).

Input: candidate sequence ϕ, desired GC-content GC_{desired}, GC tolerance ϵ, task structure part $\bar{\omega}$
Returns: improved sequence ϕ^*

$GC_\phi \leftarrow$ get_gc(ϕ)
$L_{\bar{\omega}} \leftarrow$ get_structure_loss(ϕ, $\bar{\omega}$)
absolute_gc_difference $\leftarrow |GC_{\text{desired}} - GC_\phi|$
if *absolute_gc_difference* $<= \epsilon$ **then**
  return ϕ
end
nucleotides_to_replace $\leftarrow$ get_nucleotides_to_be_replaced(GC_ϕ, GC_{desired})
replacing_nucleotides $\leftarrow$ {A, C, G, U}\ nucleotides_to_replace
positions_for_replacements $\leftarrow$ get_replacement_positions(ϕ, nucleotides_to_replace)
$\phi^* \leftarrow \phi$
while not *gc_satisfied*(ϕ^*, $GC_{desired}$, ϵ) **do**
  new_nuc $\leftarrow$ random_select(replacing_nucleotides)
  $i \leftarrow$ random_select(positions_for_replacements)   # sample without replacement
  new_candidate $\leftarrow$ replace(ϕ^*, i, new_nuc)
  $GC_{new} \leftarrow$ get_gc(new_candidate)
  if *gc_exceeded_threshold(GC_new, $GC_{desired}$)* **then**
    return ϕ^*
  end
  $L \leftarrow$ get_structure_loss(new_candidate, $\bar{\omega}$)
  if $L \leq L_{\bar{\omega}}$ **then**
    $\phi^* \leftarrow$ new_candidate
  end
end
return ϕ^*

D Riboswitch Design Space

Table S3: Originally proposed theophylline riboswitch constructs and partial RNA design space formulation. (Top) The sequence parts of the six originally proposed riboswitch constructs by Wachsmuth et al. [7] and the sequence part of the design space. (Bottom) The corresponding structure parts of the constructs and the structural part of the desiDesign Space of libLEARNA for the design of theophylline riboswitch constructs. The constructs RS1, RS2, RS3, RS4, RS8 and RS10 were proposed by (37). Highlighted regions mark parts that are shared across all the original riboswitch constructs. These regions are used to construct a design space for libLEARNA. At the bottom, the figure shows an example prediction of libLEARNA for the given design space.gn space. Red: TCT8-4 theophylline aptamer; green: variable length spacer domain; blue: domain ought to pair with the aptamer (complementary to the 3′-end of the aptamer sequence in the original design); black: 8-U-stretch. Masked positions are indicated with `?`, positions for extensions are indicated with $\overset{*}{?}$.

Construct	Aptamer	Spacer	Complementary Region	8-U-Stretch
RS1 Sequence	AAGUGAUACCAGCAUCGUCUUGAUGCCCUUGGCAGCACUUCA	UUACAUC	UGAAGUGCUGCC	UUUUUUUU
RS2 Sequence	AAGUGAUACCAGCAUCGUCUUGAUGCCCUUGGCAGCACUUCA	UGAUCUCGCU	UGAAGUGCUGC	UUUUUUUU
RS3 Sequence	AAGUGAUACCAGCAUCGUCUUGAUGCCCUUGGCAGCACUUCA	UUUACAUACUCGGUAAAC	UGAAGUGCUGCCA	UUUUUUUU
RS4 Sequence	AAGUGAUACCAGCAUCGUCUUGAUGCCCUUGGCAGCACUUCA	AACCGAAAUUUGCGCU	UGAAGUGCUGC	UUUUUUUU
RS8 Sequence	AAGUGAUACCAGCAUCGUCUUGAUGCCCUUGGCAGCACUUCA	CUCCUAGUGGAG	UGAAGUGCUG	UUUUUUUU
RS10 Sequence	AAGUGAUACCAGCAUCGUCUUGAUGCCCUUGGCAGCACUUCA	GAAAUCUC	UGAAGUGCUG	UUUUUUUU
Task Sequence Parts	AAGUGAUACCAGCAUCGUCUUGAUGCCCUUGGCAGCACUUCA	$\overset{*}{?}$??????$\overset{*}{?}$	UGAAGUGCUG$\overset{*}{?}$	UUUUUUUU
RS1 Structure	...........(((((.....)))))....((((((((((((	....	))))))))))))	....
RS2 Structure	...........(((((.....)))))....((((((((((((	....	)))))))))))	).......
RS3 Structure	...........(((((.....)))))...(((((((((((((	(((((.......))))).	)))))))))))))	....
RS4 Structure	...........(((((.....)))))....((((((((((((	(..((.......)).)	)))))))))))	).......
RS8 Structure	........((((((((.....)))))...)))((((((((((	((((....))))	))))))))))	....
RS10 Structure	........((((((((.....)))))...)))((((((((((	((....))	))))))))))	....
Task Structure Parts	........???(((((.....)))))...???((((((((((	$\overset{*}{?}$??....$\overset{*}{?}$	))))))))))$\overset{*}{?}$	?.......

E Additional Results

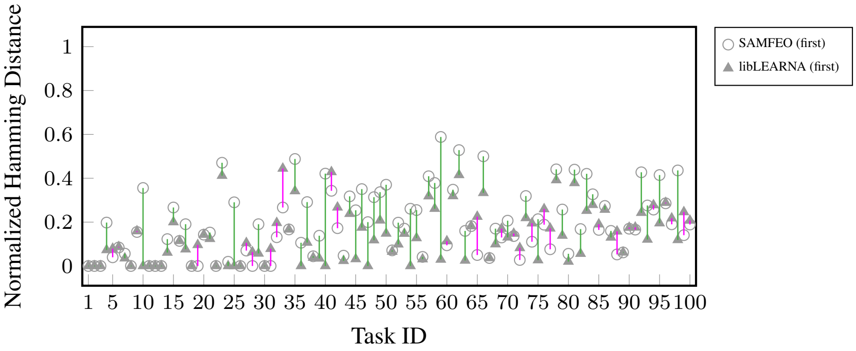

Figure S3: Comparison of the first predictions of SAMFEO [8] and libLEARNA on all tasks of the Eterna100 benchmark version 2. The plot shows the average normalized Hamming distance of the first prediction for all tasks of the benchmark. Green bars indicate tasks where libLEARNA achieves lower Hamming distance, and purple bars indicate tasks where the Hamming distance is above that of SAMFEO's prediction.

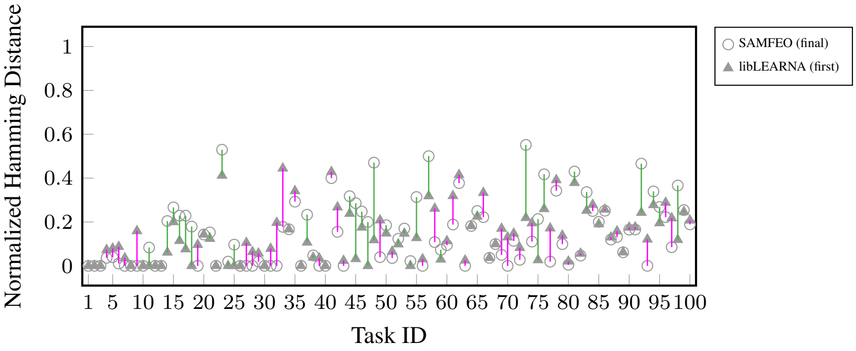

Figure S4: Comparison of the final predictions of SAMFEO [8] after 1 iteration of a full optimization and libLEARNA's first predictions on all tasks of the Eterna100 benchmark version 2. The plot shows the average normalized Hamming distance of the first prediction for all tasks of the benchmark. Green bars indicate tasks where libLEARNA achieves lower Hamming distance, and purple bars indicate tasks where the Hamming distance is above that of SAMFEO's prediction.

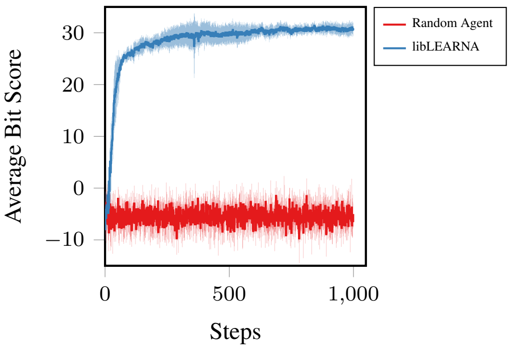

Figure S5: Average bitscore of designed candidates for the covariance model of Hammerhead Ribozyme (Type III). A step in the figure corresponds to 100 episodes.

References

[1] Sam Griffiths-Jones, Alex Bateman, Mhairi Marshall, Ajay Khanna, and Sean R. Eddy. Rfam: an RNA family database. *Nucleic Acids Research*, 31(1):439–441, 01 2003. ISSN 0305-1048. doi: 10.1093/nar/gkg006.

[2] Ronny Lorenz, Stephan H. Bernhart, Christian Höner zu Siederdissen, Hakim Tafer, Christoph Flamm, Peter F. Stadler, and Ivo L. Hofacker. Viennarna package 2.0. *Algorithms for Molecular Biology*, 6(1):26, Nov 2011. ISSN 1748-7188.

[3] Michael F Sloma and David H Mathews. Exact calculation of loop formation probability identifies folding motifs in rna secondary structures. *RNA*, 22(12):1808–1818, 2016.

[4] Rohan V Koodli, Boris Rudolfs, Hannah K Wayment-Steele, Eterna Structure Designers, and Rhiju Das. Redesigning the eterna100 for the vienna 2 folding engine. *bioRxiv*, pages 2021–08, 2021.

[5] Stefan Falkner, Aaron Klein, and Frank Hutter. BOHB: Robust and efficient hyperparameter optimization at scale. In Jennifer Dy and Andreas Krause, editors, *Proceedings of the 35th International Conference on Machine Learning*, volume 80 of *Proceedings of Machine Learning Research*, pages 1437–1446, Stockholmsmässan, Stockholm Sweden, 10–15 Jul 2018. PMLR.

[6] Frederic Runge, Danny Stoll, Stefan Falkner, and Frank Hutter. Learning to design RNA. In *International Conference on Learning Representations*, 2019.

[7] Manja Wachsmuth, Sven Findeiß, Nadine Weissheimer, Peter F. Stadler, and Mario Mörl. De novo design of a synthetic riboswitch that regulates transcription termination . *Nucleic Acids Research*, 41(4):2541–2551, 12 2012. ISSN 0305-1048.

[8] Tianshuo Zhou, Ning Dai, Sizhen Li, Max Ward, David H Mathews, and Liang Huang. Rna design via structure-aware multifrontier ensemble optimization. *Bioinformatics*, 39(Supplement_1):i563–i571, 2023.
